# Antimicrobial Resistance Profiles of Bacterial Pathogens Associated with Acute Diarrheal Disease: A Three-Year Retrospective Study in a Romanian Tertiary-Care Hospital

**DOI:** 10.3390/antibiotics15070632

**Published:** 2026-06-23

**Authors:** Alina Maria Borcan, Laura Georgiana Caravia, Bianca Secuiu, Calin Andrei Borcan, Madalina Simoiu

**Affiliations:** 1 Faculty of Medicine, The University of Medicine and Pharmacy “Carol Davila”, Dionisie Lupu Street, No. 37, 050474 Bucharest, Romania; alina.borcan@umfcd.ro (A.M.B.); laura.caravia@umfcd.ro (L.G.C.); andrei-calin.borcan0721@stud.umfcd.ro (C.A.B.); madalina.simoiu@umfcd.ro (M.S.); 2 The National Institute of Infectious Diseases “Prof. Dr. Matei Balș”, Doctor Grozovici Street, No. 1, 021105 Bucharest, Romania

**Keywords:** antimicrobial resistance, acute diarrheal disease, *Campylobacter*, *Salmonella*, fluoroquinolone resistance, macrolide resistance, gastrointestinal, eastern Europe, one health, Romania

## Abstract

Background: Despite its typically self-limiting course, acute diarrheal disease continues to be clinically relevant from an antimicrobial resistance surveillance perspective. In-depth analyses at a national level remain limited, with available Romanian studies from the last decade focusing on individual pathogens, often relying on a restricted isolate collection. In this context, we aimed to evaluate antimicrobial resistance profiles and distribution of *Salmonella* spp., *Campylobacter* spp., *Escherichia coli*, *Yersinia* spp. and *Shigella* spp. Methods: Data was obtained from records from the Microbiology Laboratory of a tertiary-care hospital serving the south region of Romania, over a 3-year period. Results: *Campylobacter* spp. had high resistance rates to ciprofloxacin (81.65% for *C. jejuni*; 85.15% for *C. coli*) and tetracycline (44.65% for *C. jejuni*; 56.07% for *C. coli*). Erythromycin resistance remained low and stable over the study period, with no statistically significant temporal variation; however, *C. coli* isolates demonstrated significantly higher erythromycin (*p* = 0.001) and tetracycline (*p* = 0.008) resistance rates compared to *C. jejuni*. Overall *Salmonella* spp. resistance rate to ciprofloxacin was 46.00%, with higher resistance observed in serogroups C (63.64%) and D (52.53%) (*p* < 0.01). Ampicillin (AMP) resistance varied significantly across years and serogroups, with serogroup B consistently demonstrating higher resistance rates (40.48%) (*p* < 0.001). *E. coli* isolates reacting with pathotype-associated O antisera revealed high resistance levels to ampicillin (41.57%), amoxicillin–clavulanic acid (AMC) (38.73%) and sulfamethoxazole–trimethoprim (SXT) (19.25%), with low resistance levels to ciprofloxacin (9.04%) and ceftriaxone (CRO) (9.71%); no significant variation in resistance patterns was identified across years or serological pools, suggesting a relatively stable resistance profile over the study period. *Yersinia* spp. isolates showed no notable antimicrobial resistance levels. *Shigella* spp. isolates exhibited high resistance for ampicillin (78.57%), sulfamethoxazole–trimethoprim (68.75%), amoxicillin–clavulanic acid (50.00%) and ceftriaxone (35.41%). Conclusions: This study addressed a recognized gap in Romanian and Eastern European surveillance data and aims to contribute to a stronger evidence base for future epidemiological investigations and antimicrobial stewardship efforts. Resistance rates identified in our study may provide valuable information for comparison with data generated from veterinary, food and environmental surveillance programs, thereby supporting a more comprehensive understanding of antimicrobial resistance (AMR) epidemiology. These findings may additionally contribute to the development of coordinated strategies aimed at mitigating the emergence and spread of AMR.

## 1. Introduction

Acute diarrheal disease (ADD) is defined by the World Health Organization (WHO) as the presence of stools of changed consistency for three or more times per day or with an increased frequency than usual [1]. It is placed as the eighth leading cause of death among all ages. In low-income countries, it stands as the second leading cause of mortality in children under 5 years, whereas, in developed countries, it can lead to multiple emergency room visits and hospital admissions. Nevertheless, fatal outcomes may still occur, as roughly 300 deaths per year are attributed to acute diarrhea in western European countries [2].

*Campylobacter* species, mainly *C. jejuni* and *C. coli*, are among the leading bacterial causes of infectious diarrhea worldwide. Initially, the genus was entirely linked to veterinary diseases in 1886, and it was not until nearly a century later that the first reports of *Campylobacter* as a human pathogen responsible for colitis appeared [3]. Campylobacteriosis is the most commonly reported gastrointestinal disease in Europe since 2005, with the highest notification rates being in Czechia and Luxembourg and lowest rates in Eastern European countries, including Romania [4]. The disease follows a self-limited course with mild symptoms; however, children, infants and immunocompromised patients may experience severe forms. Further complications through “molecular mimicry”, predominantly Guillain–Barré syndrome (GBS) and reactive arthritis, may occur [3]. GBS carries a mortality rate of 3–10%, that is triggered, most frequently, by *C. jejuni* [5]. Campylobacteriosis-associated reactive arthritis has an incidence of 8% among adults and 3% among children [6].

The decision to initiate antibiotic therapy (fluoroquinolones, macrolides, aminoglycosides and tetracyclines) is reserved for moderate and severe forms of campylobacteriosis and is influenced by patients’ status. Antibiotic resistance has been noted since the 1990s and is associated with antimicrobial use in both human and veterinary settings [3,7]. Fluoroquinolones (FQ) used to be one of the most commonly used class of antibiotics in treating this foodborne illness; unfortunately, resistance rates have significantly risen to such a degree that WHO has included fluoroquinolone-resistant *Campylobacter* on the list of high-priority pathogens for research and development of new antibiotics [8,9].

The second most common gastrointestinal infection in the European Union is salmonellosis, a disease caused by non-typhoidal *Salmonella* (NTS) serovars, that accounted for 78.307 cases in 2023, with the highest rates found in children under 5 years [10]. Despite mostly being a self-limited mild illness, in 2023, Romania was the only country in UE with a 100% hospitalized case rate of salmonellosis, even if it was not among the countries with the highest notification rates [10]. Severe complications, including bacteremia and invasive disease, may develop in young children, elderly individuals and immunocompromised patients, highlighting the clinical importance of effective antimicrobial therapy in selected cases.

Although most *Escherichia coli* strains normally reside in the colon and are the main facultative anaerobic intestinal species, playing a key role in intestinal physiology, some pathogenic strains evolved and acquired a critical set of characteristics through horizontal gene transfer, allowing them to become an important etiological agent of diarrheal diseases. These strains are grouped under the term diarrheagenic *E. coli* (DEC) [11,12]. From an epidemiological point of view, DEC are found in community-based surveillance and hospital panels from low- and middle-income countries [13]. Closely related to *E. coli*, *Shigella* species are one of the oldest human-specific pathogens, with its natural reservoirs and host being humans and primates [14]. This pathogen demonstrates high transmissibility, primarily via foodborne transmission, with infection occurring after an inoculum as low as 10–100 organisms [15,16]. Shigellosis is considered to be of higher severity, confirmed by its place as the second leading cause of diarrheal mortality, after rotavirus, in children under 5 years, amounting to 81.800 deaths, according to the 2021 Global Burden of Disease study [17]. Bacteremia is among the complications found in shigellosis, as well as reactive arthritis, hemolytic uremic syndrome and seizures [16]. Due to its highly contagious nature, antibiotic therapy is also recommended for those in close contact environments, independent from clinical severity, in order to reduce shedding duration [16]. According to the updated 2024 WHO Bacterial Priority Pathogens List, FQ-resistant *Shigella* spp. classification into the high-priority group persists, sharing this category with FQ-resistant NTS [9]. Yersiniosis is placed as the fourth most frequent gastrointestinal infection in the EU, according to the 2022 ECDC reports [18]. Generally, Europe exhibits the least prevalence of this pathogen, with most yersiniosis cases reported in Africa [19].

To provide national context, data reported for Romania in annual surveillance reports of the European Center for Disease Prevention and Control were extracted and summarized in Table 1. Between 2018 and 2023, *Campylobacter* spp. and *Salmonella* spp. accounted for the highest number of reported cases, with variability observed across years, while *Yersinia* spp. and *Shigella* spp. were reported less frequently.

European surveillance data provide a valuable framework; however, detailed analysis at a national level remains limited. Most studies conducted in the last decade have focused on individual pathogens, while comprehensive investigations evaluating antimicrobial resistance data among multiple bacterial agents associated with infectious diarrhea are scarce. Furthermore, available Romanian studies that focused on *Campylobacter* spp., *Salmonella* spp., diarrheagenic *E. coli*, *Shigella* spp. and *Yersinia* spp. in humans, alongside antimicrobial susceptibility data, are particularly limited, highlighting important gaps in national surveillance. In this context, we aimed to evaluate the distribution and antimicrobial resistance profiles of bacterial pathogens isolated from patients with ADD in Romania, to provide critical data that may support clinical decision-making, antimicrobial stewardship and future surveillance efforts. Local microbiological surveillance data remain particularly important in regions where national integrated antimicrobial resistance reporting is limited or incomplete, as resistance patterns may vary substantially across geographic settings and bacterial species. Although hospital-based retrospective datasets cannot independently establish transmission pathways or national incidence estimates, they may provide valuable insights into clinically significant resistance patterns within a defined regional setting.

## 2. Results

### 2.1. General Distribution of Bacterial Isolates

**Figure 1:**
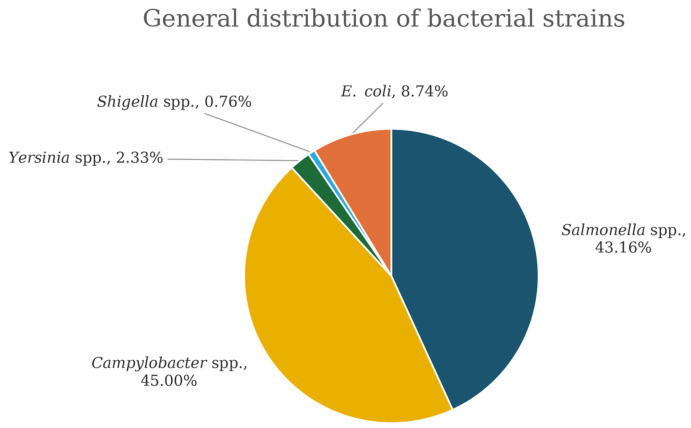
General distribution of bacterial pathogens in acute diarrheal disease, 2023–2025.

Between 2023 and 2025, a total of 2231 bacterial isolates were identified from 21,511 of stool samples, from pediatric (N = 1682; 75.39%) and adult (N = 549; 24.61%) patients, with a positivity rate of 10.37%. The median age was 3 years old (IQR:1–16; range: 0.08–90). The mean age was 13.6 years old (SD 20.3), reflecting a markedly right-skewed distribution. Among pediatric patients, 74.14% (N = 1247) were children under 5 years of age. Overall, the most common pathogen was *Campylobacter* spp. (N = 1004, 45.00%), followed by *Salmonella* spp. (N = 963, 43.16%), as seen in Figure 1.

**Figure 2:**
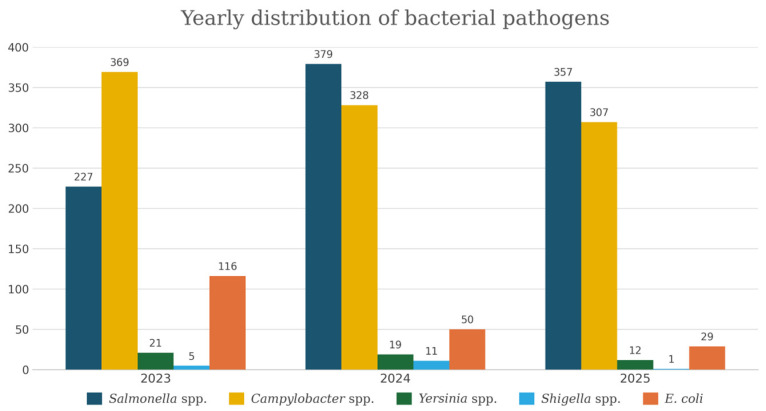
Yearly distribution of bacterial pathogens isolated from stool samples, 2023–2025.

Figure 2 below depicts the annual distribution of the pathogens. In 2023, the highest number of positive cases are attributed to *Campylobacter* spp., whereas, in the following years, the highest number of positive cases are associated with *Salmonella* spp.

We have also looked at seasonal patterns of positive bacterial samples across the three-year period (Table 2) revealing temporal variations in pathogen distribution. In 2023, higher numbers of *Campylobacter* spp. cases were observed during the summer and autumn months, with *Salmonella* spp. representing the second most frequent pathogen. In contrast, in 2024 and 2025, a shift was observed, with *Salmonella* spp. cases exceeding *Campylobacter* spp. during the summer and autumn months. Peak incidence of *Campylobacter* spp. was recorded in July and October 2023, May and June 2024, May and July 2025, with a noticeable decline in June 2025. For *Salmonella* spp., incidence peaks were observed in June and July 2023, August 2024 and July 2025.

### 2.2. Campylobacter Species

A total of 1004 samples were positive with *Campylobacter* spp., with the most common species being *C. jejuni*, accounting for 78.49% (N = 788) of the cases, followed by *C. coli*, representing 21.41% (N = 215) and *C. upsaliensis*, found in 0.10% of the cases (N = 1).

**Figure 3:**
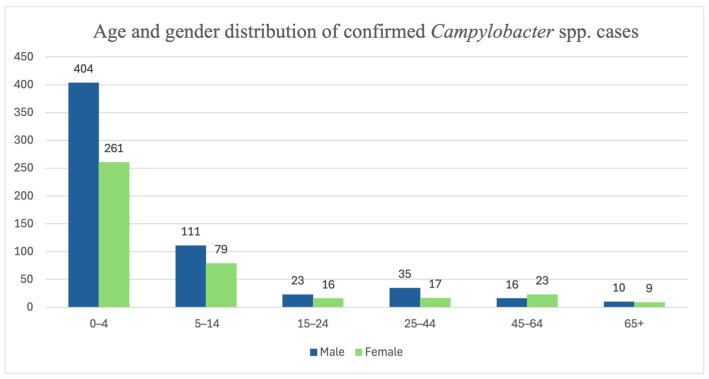
*Campylobacter* spp. cases by age and gender, 2023–2025.

Overall, 59.66% (N = 599) of the patients were males and 40.34% (N = 405) were females. Figure 3 below depicts confirmed *Campylobacter* spp. cases categorized by age and gender. The highest number of confirmed cases for both genders were found in children under 5 years of age (N= 665), out of which 404 patients were males. Moreover, given that the number of *Campylobacter* spp. cases in male patients were higher than the ones in female patients in the 0–44 age group, we further divided the cases into the following age groups: <45 years old and ≥45 years old. The distribution of cases by sex differed significantly between the two age groups ($\chi^{2}$ = 5.628, df = 1, *p* = 0.018), with a predominance of male cases among individuals younger than 45 years.

In our laboratory, routine antimicrobial susceptibility testing for *Campylobacter* spp. involved only three agents: ciprofloxacin (CIP), erythromycin (ERY) and tetracycline (TET). A total of 629 *Campylobacter jejuni* isolates had complete susceptibility results for all three antimicrobials. Of these, 2.71% (N = 17) were resistant to all tested antimicrobial classes and represent a minimum estimated prevalence of multi-drug resistance (MDR). Individually, ciprofloxacin resistance was observed in 81.65% of cases (95% CI: 78.7–84.4%; N = 614/752), with 15.69% (N = 118) of isolates being susceptible at increased exposure (I). Erythromycin susceptibility testing revealed a resistance rate of 4.47% (95% CI: 3.11–6.18%; N = 34/761). Tetracycline susceptibility testing revealed a resistance rate of 44.65% (95% CI: 40.77–48.58%; N = 288/645).

Table 3 below shows antibiotic resistance rates of *Campylobacter jejuni* strains for ciprofloxacin, erythromycin and tetracycline, for each year included in the study. Ciprofloxacin resistance differed significantly across the study period (*p* = 0.029), with the highest percentage found in 2024 (86.75%) (95% CI: 81.89–90.7%). Erythromycin resistance remained consistently low throughout the study period, with no statistically significant variation across years (*p* = 0.143). Tetracycline resistance percentage was highest in 2023 (46.13%); however, differences across years were not statistically significant (*p* = 0.513).

Similarly, for *Campylobacter coli* isolates, a total of 164 strains had complete susceptibility results for all three antimicrobials; 4.88% (N = 8) represented a minimum estimated prevalence of MDR. Individually, ciprofloxacin susceptibility testing revealed a resistance rate of 85.15% (95% CI: 79.48–89.75%; N = 172/202) with 13.37% (N = 27) of isolates being susceptible at increased exposure (I). Erythromycin susceptibility testing showed a resistance rate of 10.34% (95% CI: 6.51–15.38%; N = 21/203); Tetracycline resistance was detected in 56.07% of *C. coli* isolates (95% CI: 48.33–63.59%; N = 97/173).

Table 4 above depicts the varied annual antibiotic resistance rates of *Campylobacter coli* isolates. Ciprofloxacin resistance differed across the study period (*p* < 0.001), with the highest resistance registered in 2024 (95.71%) (95% CI: 87.98–99.11%). For erythromycin, the highest resistance rate was found in 2025 (11.67%) with no significant difference across years (*p* = 0.878). Lastly, tetracycline resistance presented a temporal variation (*p* = 0.014), with the highest percentage recorded in 2024 (68.12%) (95% CI: 55.79–78.83%).

Lastly, we wanted to compare antimicrobial resistance between the two species studied. Ciprofloxacin resistance was high in both species, with no statistically significant difference observed (81.65% for *C. jejuni* vs. 85.15% for *C. coli*, *p* = 0.248). In contrast, erythromycin resistance was higher in *Campylobacter coli* compared to *Campylobacter jejuni* (10.37% vs. 4.47%, *p* = 0.001). Similarly, tetracycline resistance was considerably higher in *C. coli* than in *C. jejuni* isolates (56.07% vs. 44.65%, *p* = 0.008). Combined resistance to ciprofloxacin and erythromycin was identified in 51 out of 936 *Campylobacter* isolates. When stratified by species, this dual resistance was observed in 9.2% (95% CI: 5.53–14.13%; N = 18/196) of *C. coli* isolates and 4.5% (95% CI: 3.08–6.20%; N = 33/740) of *C. jejuni* isolates. This difference was statistically significant (*χ*^2^ = 6.71, *p* = 0.010, Cramer’s V = 0.085).

### 2.3. Salmonella Species

*Salmonella* spp. was identified in 963 stool samples, with the most frequent serogroup being *Salmonella* serogroup D, representing 68.33% (N = 658) of the isolates, followed by serogroup B (18.48%, N = 178) and serogroup C (11.42%, N = 110). A small percentage (1.77%, N = 17) of *Salmonella* spp. isolates exhibited non-specific agglutination with multiple O antisera and was, therefore, classified as non-typeable. All tested D serogroups were negative for the Vi antigens.

**Figure 4:**
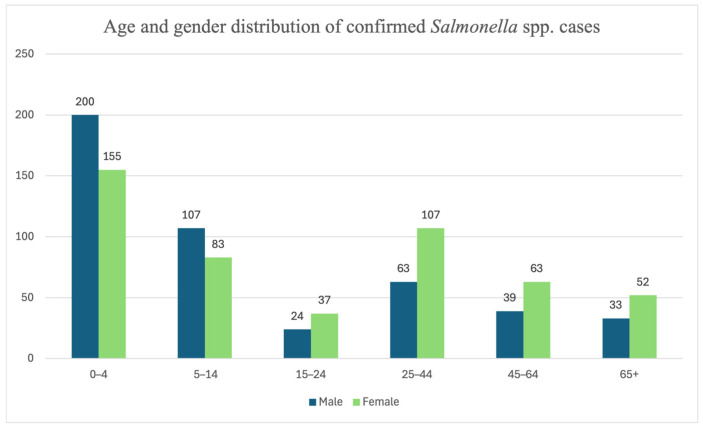
Confirmed *Salmonella* spp. cases by age and gender. 2023–2025.

Out of the 963 confirmed *Salmonella* spp. cases, 48.39% (N = 466) were male patients and 51.61% (N = 497) were female patients. Figure 4 below depicts *Salmonella* spp. cases categorized by age and gender. Highest number of confirmed cases for both genders were found in children under 5 years of age (N = 355) and the lowest number of confirmed cases in the age group 15–24 years old (N = 61). Given the observed shift in sex distribution after childhood, age groups were categorized into pediatric (<15 years) and adolescent/adults (≥15 years) for statistical analysis. A significant association was observed between sex and age categories ($\chi^{2}$ = 31.694, df = 1, *p* < 0.001). Male cases accounted for the majority of cases in the 0–14-year age group (56.3% N = 307 vs. 43.7% N = 238), whereas female patients accounted for most cases among patients aged ≥ 15 years (62.0% N = 259 vs. 38.0% N = 159).

In our laboratory, antimicrobial susceptibility testing for *Salmonella* was performed for six antimicrobials: ampicillin, ciprofloxacin, amoxicillin–clavulanic acid, cefotaxime (CTX), sulfamethoxazole–trimethoprim and ceftriaxone. A total of 959 isolates had complete susceptibility results available across all tested antimicrobial classes, and 1.36% (N = 13) represented a minimum estimated prevalence of MDR based on the antimicrobial panel routinely used in our laboratory. Ciprofloxacin susceptibility testing revealed a resistance rate of 47.92% (95% CI: 44.63–51.22%; N = 437/912), with one strain showing susceptibility at increased exposure. When it comes to third-generation cephalosporins, overall resistance rate was 1.10% (95% CI: 0.47–2.16%; N = 8/724) for ceftriaxone and 2.72% (95% CI: 0.74–6.82%; N = 4/147) for cefotaxime. Moreover, 2.72% (95% CI: 0.56–7.76%; N = 3/110) of tested isolates exhibited resistance to both cephalosporins. Ampicillin resistance rate was 11.62% (95% CI: 9.61–13.88%; N = 106/912). Lastly, after sulfamethoxazole–trimethoprim and amoxicillin–clavulanic acid susceptibility testing, overall resistance rates of 3.34% (95% CI: 2.29–4.68%; N = 32/958) and 8.79% (95% CI: 6.80–11.13%; N = 62/705), respectively, were observed.

Resistance patterns differed across *Salmonella* spp. serogroups for all antibiotics tested, as depicted in Table 5 below. Ampicillin resistance (*p* < 0.001) was the most frequent in serogroup B (40.48%) and the least frequent in serogroup D (3.65%). Similarly, resistance to amoxicillin–clavulanic acid (*p* < 0.001), was elevated in serogroup B (23.66%). For ciprofloxacin (*p* < 0.01), higher rates were observed in serogroups C (63.64%) and D (52.53%), while serogroup B exhibited a lower rate (13.64%). Furthermore, although isolates exhibited generally lower rates, resistance to second generation cephalosporins, namely cefotaxime (*p =* 0.001) and ceftriaxone (*p* = 0.02), in addition to sulfamethoxazole–trimethoprim (*p* < 0.001) also varied significantly across serogroups.

Given the overall higher resistance to ciprofloxacin and ampicillin, we decided to look more into how the resistance to these two antibiotics changed across the years, regardless of serogroups (Table 6). Ampicillin resistance differed across the study period (*p* = 0.007), whereas no statistically significant temporal variation was observed for ciprofloxacin resistance (*p* = 0.071).

### 2.4. Escherichia coli

In our laboratory, antimicrobial susceptibility testing for presumptive pathotype-associated *E. coli* was performed for five antimicrobials: ampicillin, ciprofloxacin, amoxicillin–clavulanic acid, sulfamethoxazole–trimethoprim and ceftriaxone. Among the 195 stool samples positive with *E. coli* strains reacting with pathotype-associated O antisera, 187 isolates had complete antimicrobial susceptibility data available and 8.02% (N = 15) represented the minimum estimated prevalence of MDR. Overall, antimicrobial resistance rates were 41.57% (95% CI: 34.25–49.18%; N = 74/178) for ampicillin, 9.04% (95% CI: 5.35–14.08%; N = 17/188) for ciprofloxacin, 38.72% (95% CI: 31.43–46.42%; N = 67/173) for amoxicillin–clavulanic acid, with 4.62% (N = 8) of the strains being susceptible at increased exposure, 19.25% (95% CI: 13.86–25.64%; N = 36/187) for sulfamethoxazole–trimethoprim and 9.71% (95% CI: 5.76–15.1%; N = 17/175) for ceftriaxone. No statistically significant temporal variation in antimicrobial resistance rates was identified among presumptive pathotype-associated *E. coli* isolates across the study period (*p* > 0.05 for all tested antibiotics). Approximately half of *E. coli* isolates, translating to 52.31% (N = 102), agglutinated with Pool 1, which contains O26, O103, O111, O145, O157 antigens, followed by 30.77% (N = 60) of strains that agglutinated with Pool 2, containing O55, O119, O125ac, O127, O128ab antigens, and, 16.92% (N = 33) of strains that agglutinated with Pool 3, containing O86, O114, O121, O126, O142. Minor variations in antimicrobial resistance rates were observed among isolates agglutinating with different polyvalent O antisera pools; however, no significant variation in resistance patterns was identified across serological pools (*p* > 0.05 for all comparisons).

### 2.5. Yersinia and Shigella Species

A total of 52 cases were positive with *Yersinia* spp., all of them pertaining to *Y. enterocolitica* species, out of which 1.92% (95% CI: 0.04–10.26%; N = 1/52) were resistant to ciprofloxacin, with one isolate being susceptible at increased exposure. Sulfamethoxazole–trimethoprim susceptibility testing revealed a resistance rate of 1.96% (95% CI: 0.04–10.45%; N = 1/51). All 46 strains that were tested for ceftriaxone susceptibility were found to be susceptible. The limited panel of antibiotics tested did not allow classification according to MDR criteria.

Our study found 17 *Shigella* spp. positive cases across the study period. Most of them were positive with *S. sonnei* (N = 14), followed by *S. boydii* (N = 2), and *S. flexneri* (N = 1). Overall, 5.88% (95% CI: 0.14–28.69%; N = 1/17) of the strains were resistant to ciprofloxacin, with three isolates being susceptible at increased exposure. Out of the *Shigella* spp. isolates that were tested for ampicillin and ceftriaxone susceptibility, 78.57% (95% CI: 49.2–95.34%; N = 11/14) and 35.71% (95% CI: 12.76–64.86%; N = 5/14), respectively, were resistant. Moreover, resistance to sulfamethoxazole–trimethoprim and amoxicillin–clavulanic acid was observed in 68.75% (95% CI: 41.34–88.98%; N = 11/16) and 50.00% (95% CI: 24.65–75.35%; N = 8/16) of the isolates, respectively.

## 3. Discussion

In this study, we analyzed the common bacterial pathogens in ADD, with a focus on antimicrobial resistance in a tertiary-care hospital serving the south region of Romania. Overall, the most common bacterial pathogen found in our stool samples from 2023 to 2025 was *Campylobacter* spp. This finding was somewhat unexpected in the national context, as annual ECDC reports indicate that, in Romania, *Salmonella* spp. cases outnumber *Campylobacter* spp. cases (Table 1), despite *Campylobacter* spp. representing the leading reported bacterial pathogens associated with ADD at the European level [4]. However, annual distribution of cases and seasonal patterns in our study show that *Salmonella* spp. cases surpass *Campylobacter* spp. cases in 2024 and 2025, with summer peaks being dominated by *Salmonella* spp. during these two years. A significant male predominance was observed among *Campylobacter* spp. cases occurring before the age of 45 years, whereas a more balanced sex distribution was observed in older patients. The reasons underlying these age- and sex-related differences cannot be determined from the present dataset; however, similar sex-associated patterns have been reported in large international studies investigating *Campylobacter* spp. epidemiology, suggesting that such observations are not unique to our population. The mechanisms responsible for these differences remain incompletely understood and are likely multifactorial [20,21]. In contrast, *Salmonella* spp. cases demonstrated a distinct age-dependent sex distribution, with males accounting for the majority of cases during childhood (0–14 years), whereas females represented most cases from adolescence onwards. A similar distribution has previously been reported in a pooled analysis of national surveillance data from eight countries, which identified higher incidence rates of *Salmonella* spp. infections among male patients during childhood and higher incidence rates among female patients in adulthood [22]. Although the reasons underlying these differences cannot be determined from our study, the consistency of this pattern across different populations may suggest that age- and sex-related factors may contribute to the epidemiology of *Salmonella* spp. infections.

*Campylobacter jejuni* was the most frequently identified bacterial pathogen among *Campylobacter* spp. isolates in our study, although both *C. jejuni* and *C. coli* share a similar pattern of antibiotic resistance for ciprofloxacin. Mohan et al. [23] described a potential ongoing horizontal gene transfer between the two *Campylobacter* species, after studying the overall distribution of AMR-conferring alleles. Ciprofloxacin resistance rates were comparably high in both *C. coli* and *C. jejuni*, with no significant difference. While our data does not allow assessment of the underlying genetic mechanisms, these findings may support and are consistent with previous reports describing the dissemination of antimicrobial resistance determinants across *Campylobacter* species. According to the fourth joint inter-agency report on integrated analysis of antimicrobial consumption and the occurrence of antimicrobial resistance in bacteria from humans and food-producing animals (JIACRA), in 2021, the EU/EEA population-weighted mean consumption of FQ and other quinolones was 6.3 mg/kg estimated biomass in humans and 2.9 mg/kg of estimated biomass in food-producing animals. The report also highlights a significant positive association between consumption of FQ in food-producing animals and FQ resistance in *C. jejuni* from humans, although univariate analysis could not identify the same for *C. coli*, where consumption of FQ in pigs was only associated with FQ resistance in pigs, not humans [24,25]. Regarding ciprofloxacin-resistant *C. jejuni*, the high resistance rate we found in our study (81.65%) is comparable to findings of a previous Romanian study [7], where FQ resistance rate was 74.7%, and consistent with reported high resistance rates of neighboring countries: 88.6% in Hungary, 91% in Poland and 93.3% in Bulgaria [26]. Moreover, studies done on broiler chickens in Romania have found FQ resistance rates varying from 79% to 96.5%, with similarly high resistance rates in Hungary (89.7%), Poland (93.9%) and Bulgaria (82%) [26,27]. The similarity of FQ resistance rates reported in human and poultry isolates across Romania and neighboring Eastern European countries highlights the value of integrated One Health surveillance approaches that combine data from human, animal and environmental sectors. While the present hospital-based dataset cannot establish transmission routes or identify the sources of resistant strains, the consistently high resistance rates observed across multiple surveillance settings underscore the importance of continued monitoring of FQ resistance. This is particularly relevant given the clinical importance of FQ in the treatment of severe *Campylobacter* spp. infections and the potential public health implications of the persistence and dissemination of resistant strains [28].

Yet even though erythromycin resistance remained low and stable over the study period, with no statistically significant temporal variation, *C. coli* isolates demonstrated considerably higher rates for this antibiotic compared to *C. jejuni*. This finding aligns with previous reports suggesting interspecies differences in macrolide resistance [29,30]. The resistance rate found in our study (10.37%) was slightly higher than that reported in recent European surveillance data from 2022 (7.8%) [4] and from the EFSA (6.7%) [26] in 2023. Locally, the European Union summary report on antimicrobial resistance in zoonotic and indicator bacteria from humans, animals and food in 2022–2023, conducted by both the EFSA and ECDC, has no available data for Romania in 2023, with Hungary being the only Eastern European country reporting a 0.3% resistance rate; however, our results are comparable with rates from Greece (10%), Italy (15.4%) and Germany (11.8%) [26]. Interestingly, a Romanian study conducted between 2018 and 2024 reported complete susceptibility to erythromycin among tested *Campylobacter* spp. isolates, in contrast to our study, which identified a low percentage of erythromycin-resistant strains between 2023 and 2025. Although resistance levels remain low, this finding highlights the dynamic nature of AMR and underscores need for continuous surveillance of macrolide susceptibility among Romanian *Campylobacter* isolates [31]. Among *C. coli* isolates, the only statistically significant association identified by JIACRA between antibiotic consumption in food-producing animals and corresponding resistance in humans appears to involve macrolide use in pigs. In the EU/EEA, population-weighted mean consumption of macrolides was reported at 6.2 mg/kg estimated biomass in humans and 7.8 mg/kg of estimated biomass in food-producing animals [25]. In Romania, ERY resistance was detected in 8.3% of local porcine *C. coli* isolates, while studies from Germany and Poland reported resistance levels between 3 and 15% [25,32,33]. These observations provide relevant epidemiological context for interpreting resistance patterns in human isolates. Considering that Romania imports nearly 80% of its pork, primarily from Germany, Poland, Spain and Hungary, integrated European surveillance across humans and food-producing animals remains important for understanding the broader epidemiology of macrolide resistance [34].

Moreover, *C. coli* demonstrated a significantly higher prevalence of combined resistance to ciprofloxacin and erythromycin compared to *C. jejuni*, in agreement with the literature reports [4,29].

More than half (56.07%) of *C. coli* strains showed resistance to tetracycline in our study, consistent with data from neighboring Eastern European countries, namely Hungary (48.6%) and Slovakia (53.5%) [26]. Our observed resistance rate was below European levels reported in 2022 (71.2%) [4] and 2023 (68.2%) [26]. Regarding *C. jejuni*, the resistance rate to tetracycline found across the study period (44.65%) is similar to European levels reported in 2022 (46.6%) [4] and 2023 (47.9%), as well as those reported in neighboring countries: Bulgaria (64.7%), Hungary (47.1%) and Slovakia (52.3%). No available data was found in this report for either *Campylobacter* species for Romania between 2021 and 2023; however, a Romanian study conducted between 2017 and 2020 found a 55% resistance rate [26]. Additionally, EFSA, together with ECDC, reported statistically significant associations between tetracycline consumption in food-producing animals (poultry) and tetracycline resistance in *C. jejuni* from humans; moreover, regional datasets consistently placed Eastern European countries among the higher resistance regions for tetracycline-resistant *Campylobacter* spp., frequently exceeding 50–70% resistance rates in poultry isolates [26]. Furthermore, tetracycline resistance has also been reported among Campylobacter isolates recovered from poultry in Romania, where, unlike the pork sector, production has largely reached self-sufficiency in recent years [35]. Popa et al. [27] reported a TET resistance rate of 49.5% in *C. jejuni* isolates from slaughtered broiler chicken in North-Western Romania. While this present study does not permit assessment of transmission pathways between animal and human reservoirs, the occurrence of elevated tetracycline resistance in both human and poultry-associated isolates underscores the importance of integrated surveillance efforts encompassing all sectors.

Resistance to all three antimicrobials tested (CIP + ERY + TET) was observed in 2.70% of *C. jejuni* strains and 4.88% of *C. coli* strains. From a clinical perspective, macrolides and fluoroquinolones remain the first-line treatment for severe *Campylobacter* spp. infections; however, the rising frequency of combined resistance observed in *Campylobacter* spp. isolates raises concerns regarding the potential for reduced therapeutic efficacy, particularly in settings where *C. coli* prevalence is increasing [23]. Overall, the multitude of factors potentially contributing to AMR in *Campylobacter* spp. further emphasizes the importance of an integrated One Health approach, incorporating coordinated surveillance of antimicrobial use and resistance across humans, food-producing animals and environmental sectors [36,37].

The second most frequently identified bacterial pathogen in our study was *Salmonella* spp., with a little over two thirds belonging to serogroup D, consistent with previous Romanian studies reporting a predominance of this serogroup [38]. Additionally, European surveillance data frequently identifies serovars associated with serogroup D, particularly *Salmonella enterica* serovar Enteritidis, among the most commonly reported causes of human *Salmonella* serovars in human infections, although such associations remain presumptive in our dataset due to the absence of serovar-level identification [39]. Serogroup B represented the second most prevalent serogroup in our study, in agreement with previous Romanian findings [38]. Moreover, recent ECDC surveillance reports highlight increasing numbers of human infections caused by serogroup B serovars, particularly *Salmonella* Infantis [10].

In our laboratory, antimicrobial susceptibility testing for *Salmonella* was performed for six antibiotics (AMP, CIP, AMC, SXT, CTX and CRO) reflecting local diagnostic practices and empirical treatment choices. However, the lack of harmonized data limits comparisons with European surveillance data. Multidrug resistance, defined as resistance to at least one antibiotic from three or more antimicrobial classes, was detected in a small percentage, although interpretation is limited by the restricted antibiotic panel tested, which may have led to an underestimation of the true burden of MDR. Even so, FQ resistance continues to stand out, as, according to a global study conducted between 1991 and 2023, on 208.233 *Salmonella* genomes, from human, animals and environment samples noted that, despite regional differences, the overall trend of FQ resistance continues to be alarming, to say the least [40]. Our study identified a ciprofloxacin resistance rate of 46% among *Salmonella* spp. isolates, exceeding the overall European resistance levels reported (21.8%) [10]. Several factors may contribute to this observation, including the tertiary-care setting of the study, which may overrepresent more severe or previously treated cases. In contrast, antimicrobial acceptability interpretation was performed according to the same EUCAST criteria throughout the study period, with no breakpoint revisions for ciprofloxacin during this time, making interpretive changes unlikely to account for the observed resistance rates. Nevertheless, similar elevated resistance rates have been described in several Eastern European countries, including Poland (43.2%), Slovakia (33.1%), Hungary (31.8%), in addition to Romania (31.5%) [26]. These findings are further supported by recent Romanian studies that report ciprofloxacin resistance rates of 47.9% and 63.2%, respectively, suggesting the persistent circulation of FQ-resistant *Salmonella* strains within the region [38,41]. Ciprofloxacin resistance significantly varied across serogroups, with serogroup C exhibiting the highest resistance rate, followed by serogroup D, mirroring findings of a similar Romanian study [38]. Although serogroup-level differences were identified, the absence of serovar-level and molecular characterization precluded assessment of the potential contribution of clonal dissemination of resistant strains.

Resistance to ampicillin was detected in 11.62% of the tested isolates, below the European resistance rate reported in 2023 (21.3%), but comparable with resistance levels found in the same year in Romania (13.7%) and neighboring countries: 19.3% in Hungary, 14% in Bulgaria, 8.3% in Poland [26]. Across the study period, we found a statistically significant temporal and serogroup variation in ampicillin resistance, with serogroup B consistently demonstrating higher resistance rates, supporting previous Romanian reports [38]. Such variability underscores the importance of considering serogroup distribution when interpreting resistance data. Cephalosporin resistance was low, with 1.10% of *Salmonella* spp. isolates resistant to ceftriaxone and 2.72% of isolates resistant to cefotaxime, in accordance with reported European low levels, including Eastern European levels [26]. Overall, rising rates of antimicrobial resistance in the treatment of *Salmonella* spp. infections have increasingly been associated with treatment failure and poorer clinical outcomes, highlighting the need for continued surveillance and antimicrobial stewardship [42].

Susceptibility testing of *E. coli* strains reacting with pathotype-associated O antisera revealed high resistance rates to ampicillin, amoxicillin–clavulanic acid and sulfamethoxazole–trimethoprim, in a decreasing order, showing similarity to resistance patterns previously described in studies of molecularly confirmed diarrheagenic *E. coli* isolates [13]. Given the ability of these organisms to colonize the perineal region and ascend the urinary tract, antimicrobial resistance observed in stool samples should not be overlooked, as they may reflect clinically relevant resistance in uropathogenic strains. Moreover, the intestinal microbiota may serve as a reservoir for resistant bacteria, impacting treatment response. This is particularly relevant in women, who are at increased risk of urinary tract infections due to anatomical factors [43]. Low resistance levels were found against ciprofloxacin and ceftriaxone. Overall, we found a low percentage of minimum estimated prevalence of MDR isolates.

The least common bacterial isolates found were *Yersinia enterocolitica* and *Shigella* spp. *Yersinia enterocolitca* isolates showed no notable antimicrobial resistance levels. However, that was not the case for *Shigella* spp. isolates. Most of the strains were identified as *S. sonnei*, consistent with European surveillance data [15] and, overall, the highest resistance was observed against ampicillin, ceftriaxone, sulfamethoxazole–trimethoprim and amoxicillin–clavulanic acid. This resistance profile has substantial clinical implications, as commonly used first-line agents, such as sulfamethoxazole–trimethoprim, may have reduced effectiveness, and therefore careful selection of empirical therapy is necessary [15]. Ampicillin is no longer used as empiric treatment but can be recommended if the strain is susceptible [44]. A 2025 global report conducted by World Health Organization identified ciprofloxacin-resistant *Shigella* spp. as exhibiting the highest median annual increase antimicrobial resistance between 2018 and 2023 [45]; despite this, our findings indicate a low resistance rate.

After reviewing internationally published Romanian studies, we found a notable scarcity of studies done in the last decade investigating antimicrobial resistance among bacterial enteropathogens found in our study, isolated from human cases. To the best of our knowledge, only two studies have reported antimicrobial susceptibility data for human *Campylobacter* isolates and three for *Salmonella* isolates, as revealed in Table 7 below. Furthermore, we could not find any recent Romanian studies reporting antimicrobial resistance data for human isolates of *Shigella* spp. or *Yersinia* spp. during this set timeframe.

Available Romanian studies from the last decade focus on individual pathogens, often relying on a restricted isolate collection. This fragmented evidence base complicates the assessment of national resistance trends and hinders direct comparisons across pathogens and time periods. By simultaneously evaluating multiple clinically relevant enteric bacteria over a recent study period, our investigation provided an updated overview of AMR profiles among bacterial pathogens associated with infectious diarrhea.

The findings of the present study should also be considered within the broader One Health framework, which recognizes the interconnected roles of human, animal, food and environmental reservoirs in the emergence and dissemination of antimicrobial resistance. Consequently, monitoring antimicrobial susceptibility among human clinical isolates represents an important component of integrated resistance surveillance. This hospital-based dataset may contribute to One Health-oriented surveillance, but it cannot independently establish transmission routes or sources of resistant strains. Nevertheless, the resistance rates identified in our study may potentially provide valuable information for comparison with data generated from veterinary, food and environmental surveillance programs, thereby supporting a more comprehensive understanding of antimicrobial resistance epidemiology. The integration of such datasets may ultimately facilitate the development of coordinated strategies aimed at mitigating the emergence and spread of AMR across sectors.

## 4. Materials and Methods

### 4.1. Study Design and Data Storage

This is a retrospective study conducted between 1 January 2023 and 31 December 2025, with data obtained from records from the Microbiology Laboratory of the National Institute of Infectious Diseases “Prof. Dr. Matei Balș”, a mono-disciplinary tertiary-care hospital in Bucharest, Romania. The strains included in the study were isolated from both inpatients and outpatients of all ages, from our hospital wards and the Intensive Care Unit (ICU). Informed consent was secured from each patient or their legal guardian upon hospital admission, with data subsequently anonymized. Over the 3-year period, a total of 2231 non-duplicate pathogenic bacterial strains were isolated from stool samples. Organization, storage and data analysis was performed using Microsoft Excel version 17.0 (2024).

### 4.2. Statistical Analysis

Statistical analyses were performed using IBM SPSS Statistics version 31 (IBM Corp., Armonk, NY, USA). Categorical variables were summarized as frequences and percentages and compared using Pearson’s chi-square test. Continuous variables were described using means and standard deviations (SD) or medians and interquartile ranges (IQR), depending on data distribution. Antimicrobial resistance proportions were reported together with 95% confidence intervals (95% CI) calculated using the exact binomial (Clopper–Pearson) method in OpenEpi version 3.01 (Open Source Epidemiologic Statistics for Public Health, Atlanta, GA, USA). A two-sided *p*-value < 0.05 was considered statistically significant.

### 4.3. Bacterial Culture

Stool samples collected from patients admitted to our hospital were sent to the laboratory and underwent macroscopic evaluation. Only stool samples of changed consistency were accepted and processed, with the exception of infants and children with post-diarrheic haemolytic uremic syndrome. The specimen was inoculated on Hektoen Enteric Agar (HEKT, bioMérieux S.A., Marcy-l’Etoile, France) for *Shigella* spp. suspicion, Yersinia Selective Agar (YER, bioMérieux S.A., Marcy-l’Etoile, France) for *Yersinia* spp. suspicion, Campylosel Agar (CAM, bioMérieux S.A., Marcy-l’Etoile, France) for *Campylobacter* spp. suspicion, Sorbitol MacConkey (SMAC, bioMérieux S.A., Marcy-l’Etoile, France) for EHEC/STEC suspicion, chromID^®^ Vibrio (VIB, bioMérieux S.A., Marcy-l’Etoile, France) for *Vibrio* spp. suspicion, and/or lactose agar (CLED, ThermoScientific™—Oxoid, Wesel, Germany) for EPEC suspicion. EPEC was considered pathogenic solely in children under 2 years old, as per international recommendations. For *Salmonella* spp. processing, the specimen was first inoculated on Selenite F Broth tubes which were subsequently incubated aerobically for 24 h at 37 °C and subcultured on chromID^®^ Salmonella Elite (SALM, bioMérieux S.A., Marcy-l’Etoile, France). All inoculated plates were incubated for 18–24 h at 37 °C in an aerobic atmosphere, with the exception of Campylosel Agar (CAM), which was incubated for 48 h at 42 °C.

### 4.4. Strain Identification

Bacterial strains were identified using Matrix-Assisted Laser Desorption Ionization Time-of-Flight Mass Spectrometry (MALDI-TOF MS) Biotyper (Bruker Daltonik GmbH, Bremen, Germany) and MALDI-TOF VITEK^®^ MS PRIME (bioMérieux S.A., Marcy-l’Etoile, France) software version 1.1, VITEK^®^MS IVD Database version 3.3.

### 4.5. Antimicrobial Susceptibility Testing (AST)

AST was performed using the MICRONAUT-S Gram-negative (customized plate) Romania GN 2 EUCAST (Bruker Daltonik GmbH, Bremen, Germany) followed by interpretation according to the EUCAST (European Committee on Antimicrobial Susceptibility Testing) guidelines versions 13.1 (2023), 14.0 (2024) and 15.0 (2025), respectively. Antimicrobial susceptibility testing results were interpreted according to EUCAST criteria, applying the definition of “I” as susceptible at increased exposure. For Enterobacterales, Escherichia coli ATCC 25922 was used as quality control. For *Salmonella* spp. strains, ciprofloxacin susceptibility was defined as minimum inhibitory concentration (MIC) ≤ 0.06 mg/L (susceptible) and >0.06 mg/L (resistant). For Enterobacterales other than *Salmonella* spp., CIP susceptibility was interpreted as susceptible (S) at MIC ≤ 0.25 mg/L, resistant (R) at MIC > 0.5 mg/L and susceptible at increased exposure (I) at MIC values between these thresholds. Pefloxacin disk diffusion testing was performed simultaneously as a surrogate marker for fluoroquinolone susceptibility, in accordance with EUCAST recommendations. AMP susceptibility was interpreted as susceptible (S) at MIC ≤ 8 mg/L, resistant (R) at MIC > 8 mg/L. For AMC, susceptibility was interpreted as susceptible (S) at MIC ≤ 8 mg/L and resistant (R) at MIC > 8 mg/L, with concentration of clavulanic acid fixed at 2 mg/L. CTX and CRO susceptibility was interpreted as susceptible (S) at MIC ≤ 1 mg/L, resistant (R) at MIC > 2 mg/L and susceptible at increased exposure (I) at MIC values between these thresholds. SXT susceptibility was interpreted as susceptible (S) at MIC ≤ 2 mg/L, resistant (R) at MIC > 4 mg/L and susceptible at increased exposure (I) at MIC values between these thresholds. The EUCAST epidemiological cut-off values (ECOFFs) used for interpretation were consistent across all EUCAST versions used during the study period.

Antimicrobial susceptibility testing of *Campylobacter* spp. was carried out using the disk diffusion method: bacterial suspensions adjusted to a 0.5 McFarland standard were inoculated onto Mueller–Hinton + 5% defibrinated horse blood and 20 mg/L β-NAD agar plates (Thermo Fisher Scientific^TM^, Oxoid, Wesel, Germany). Next, three antimicrobial disks were applied: ciprofloxacin (5 μg), erythromycin (15 μg) and tetracycline (30 μg). The selected antimicrobial agents reflected the routine susceptibility testing panel used in our laboratory for *Campylobacter* spp. isolates for surveillance purposes, clinical management of campylobacteriosis and EUCAST susceptibility testing practices. Plates were incubated at 42 °C under microaerobic conditions and examined after 24 h. Plates with insufficient growth after 24 h incubation were reincubated immediately and inhibition zones were read after a total of 40–48 h incubation. Inhibition zone diameters were measured in millimeters and interpreted according to EUCAST criteria: For CIP, inhibition zone diameters ≥ 50 mm were classified as susceptible (S), inhibition zone diameters < 26 mm were classified as resistant (R) and inhibition zone diameters between ≥ 26 mm and < 50 mm were classified as susceptible at increased exposure (I). For TET, inhibition zone diameters ≥ 30 mm were interpreted as susceptible (S) and inhibition zone diameters < 30 mm were classified as resistant (R). For ERY, inhibition zone diameters ≥ 20 mm were interpreted as susceptible (S) and inhibition zone diameters < 20 mm were classified as resistant (R) for *C. jejuni*; for *C. coli*, inhibition zone diameters ≥ 24 mm were classified as susceptible (S) and inhibition zone diameters < 24 mm were interpreted as resistant (R). *Campylobacter jejuni* ATCC 33560 was used for quality control. The EUCAST epidemiological cut-off values (ECOFFs) used for interpretation were consistent across all EUCAST versions used during the study period.

Resistance rates were calculated using isolates categorized as resistant (R) as the numerator and the total number of tested isolates, represented by the sum of resistant (R), susceptible (S) and susceptible, increased exposure (I) as the denominator, in accordance with EUCAST recommendations for AMR surveillance. Strains were classified as multidrug-resistant (MDR), according to international consensus, defined as acquired non-susceptibility to at least one antimicrobial agent in three or more antimicrobial categories. [47] MDR classification was performed only for isolates with sufficient available antimicrobial susceptibility data across multiple antimicrobial classes. Due to differences in routinely tested and reported antimicrobial panels, MDR assessment was not feasible for all isolates or bacterial species. The larger denominators found in individual resistance rates reflect additional isolates for which susceptibility testing was available for one or two agents but not all three. This occurred occasionally due to limitations in antimicrobial disk availability during the study period.

### 4.6. Serogrouping

Our laboratory screens for DEC groups using commercially available polyvalent O antisera (SSI Diagnostica, Hillerød, Denmark). Colonies with morphological characteristics compatible with *Escherichia coli* on CLED agar were selected for serological characterization. Identification of isolates reacting with diarrheagenic *E. coli* O antigens was performed by slide agglutination using the SSI polyvalent antisera Pools 1, 2 and 3. The OK O Pool 1 EPEC/VTEC/STEC antisera reacts with antigens O26, O103, O111, O145, O157; OK O Pool 2 EPEC antisera with antigens O55, O119, O125ac, O127, O128ab; OK O Pool 3 EPEC antisera with antigens O86, O114, O121, O126, O142. Even though *Escherichia coli* O111 and *Escherichia coli* O26 serogrouping is included in Pool 1, we also used mono-specific OK antisera when Shiga toxin-producing *Escherichia coli* O111 and *Escherichia coli* O26 was strongly suspected. Molecular confirmation of pathotype-specific virulence genes was not available. Given the clinical nature of stool diagnostics, *Salmonella* isolates were also identified to the serogroup level, according to the White–Kauffman–Le Minor scheme, described in the WHO reference manual (9th edition, 2007) and its subsequent published supplements, as serovar identification was beyond the scope of our routine diagnostics. We used *Salmonella* antisera (SSI Diagnostica, Hillerød, Denmark) O Group Pool OMA, O:2, O:4, O:7, O:9. Identification of the Vi capsular antigen was performed using the *Salmonella* Vi monoclonal antibody (SSI Diagnostica, Hillerød, Denmark).

## 5. Conclusions

Despite its typically self-limiting course, acute diarrheal disease continues to be clinically relevant from an antimicrobial resistance surveillance perspective. Our findings highlight that antimicrobial resistance among enteric bacterial pathogens remains a dynamic and evolving challenge. The high rates of fluoroquinolone resistance observed among *Campylobacter* spp. and *Salmonella* spp., together with pathogen-specific differences such as variable resistance among *Salmonella* serogroups and the increased erythromycin resistance detected in *C. coli* in comparison with *C. jejuni*, demonstrate the need for continued monitoring of resistance patterns at both species and subtype levels. This study addressed a recognized gap in Romanian and Eastern European surveillance data and aims to contribute to a stronger evidence base for future epidemiological investigations and antimicrobial stewardship efforts and integrated surveillance strategies targeting the emergence and spread of AMR.

## 6. Limitations

This study has several limitations that should be considered when interpreting its findings. Identification of *Salmonella* spp. was limited to the serogroup level, precluding differentiation between strains with distinct pathogenic and resistance profiles. An additional limitation concerns the characterization of *E. coli* strains. Classification was based on serological reactivity with SSI polyvalent O antisera pools without molecular confirmation of virulence-associated genes. Consequently, isolates should be interpreted as presumptive serogroup-associated *E. coli*. These limitations reduce direct comparability with European surveillance data and therefore trends and strain-specific epidemiological dynamics should be interpreted cautiously. Furthermore, the relatively low MDR estimation may underestimate the true burden, as resistance characterization was based on a restricted antimicrobial testing panel. The low number of *Shigella* spp. and *Yersinia* spp. isolates limits the reliability of the conclusions for these pathogens. *Clostridioides difficile* was not assessed due to the lack of routine diagnostic testing, potentially underestimating the burden of bacterial diarrheal disease. The primary objective of the present study was descriptive microbiological surveillance rather than identification of independent predictors of antimicrobial resistance phenotypes; thus, no multivariable analyses were performed. Furthermore, multiple subgroup comparisons were conducted across years, pathogens, serogroups and antimicrobial agents and therefore, the possibility of inflated type-I error rates should be considered when interpreting statistically significant findings. In addition, the dataset used in this study pertains to a tertiary-care hospital which serves the southern region of Romania and may overrepresent more severe and complicated cases. Consequently, the observed antimicrobial resistance rates, particularly among *Campylobacter* spp. and *Salmonella* spp., may be higher than those encountered in community-based settings and could limit the representativeness of community incidence and national distribution. Despite these limitations, the study provides relevant insight into local antimicrobial resistance patterns among common bacterial pathogens associated with acute diarrheal disease.

## Figures and Tables

**Table 1:** Reported cases of *Campylobacter* spp., *Salmonella* spp., *Yersinia* spp., *Shigella* spp. and Shiga toxin-producing *E. coli* (STEC) in Romania, 2018–2023, according to annual epidemiological reports published by the European Center for Disease Prevention and Control; NA = not available.

	*Campylobacter* spp.	*Salmonella* spp.	*Yersinia* spp.	*Shigella* spp.	STEC
2018	573	NA	22	147	NA
2019	805	1383	36	117	36
2020	300	408	6	15	14
2021	348	518	15	17	6
2022	525	1010	14	25	28
2023	NA	1388	NA	NA	41

**Table 2:** Monthly distribution of bacterial isolates identified from stool samples of patients with acute diarrheal disease, 2023–2025.

Pathogen	Year	Jan	Feb	Mar	Apr	May	Jun	Jul	Aug	Sep	Oct	Nov	Dec
*Campylobacter* spp.	2023	11	22	11	25	32	37	52	44	41	48	16	30
	2024	16	21	15	27	42	44	27	32	34	37	24	9
	2025	26	19	15	24	37	12	47	29	20	29	31	18
*Salmonella* spp.	2023	17	10	7	10	13	32	34	25	27	17	16	19
	2024	6	17	19	27	36	39	49	59	39	40	22	26
	2025	25	21	17	28	33	35	57	41	42	27	19	12
*E. coli*	2023	5	7	9	6	11	2	15	14	22	10	8	7
	2024	3	7	4	1	2	2	6	9	4	7	2	3
	2025	3	5	2	0	4	0	1	3	1	5	0	5
*Yersinia* spp.	2023	4	0	0	0	2	0	5	1	1	2	2	4
	2024	3	1	3	1	3	2	1	0	2	2	1	0
	2025	6	1	1	0	1	0	0	0	2	1	0	0
*Shigella* spp.	2023	0	0	0	0	0	0	0	0	1	3	0	1
	2024	2	1	0	0	0	0	0	0	4	2	1	1
	2025	0	0	0	0	0	0	0	0	0	1	0	0

**Table 3:** Annual antimicrobial resistance rates among *C. jejuni* isolates, expressed as number of resistant isolates relative to the total number of tested isolates for each antimicrobial agent.

Antimicrobials	Number of Resistant Isolates/Total Investigated Isolates in the Study Years (%)		
	2023	2024	2025
Ciprofloxacin	231/292 (79.11%)	216/249 (86.75%)	167/211 (79.15%)
Erythromycin	11/292 (3.77%)	16/241 (6.64%)	7/228 (3.07%)
Tetracycline	131/284 (46.13%)	109/241 (45.23%)	48/120 (40.00%)

**Table 4:** Annual antimicrobial resistance rates among *C. coli* isolates, expressed as the number of resistant isolates relative to the total number of tested isolates for each antimicrobial agent.

Antimicrobials	Number of Resistant Isolates/Total Investigated Isolates in the Study Years (%)		
	2023	2024	2025
Ciprofloxacin	62/76 (81.58%)	67/70 (95.71%)	43/56 (76.79%)
Erythromycin	8/76 (10.53%)	6/67 (8.96%)	7/60 (11.67%)
Tetracycline	32/73 (43.84%)	47/69 (68.12%)	18/31 (58.06%)

**Table 5:** Antimicrobial resistance rates among *Salmonella* spp. serogroups B, C and D, expressed as the number of resistant isolates relative to the total number of tested isolates for each antimicrobial agent.

Antimicrobials	Number of Resistant Isolates/Total Investigated Isolates (%)		
	Serogroup B	Serogroup C	Serogroup D
Ciprofloxacin	24/176 (13.64%)	70/110 (63.64%)	342/651 (52.53%)
Ampicillin	68/168 (40.48%)	14/104 (13.46%)	23/630 (3.65%)
Cefotaxime	1/27 (3.70%)	4/28 (14.29%)	0/95 (0.00%)
Ceftriaxone	2/134 (1.49%)	3/85 (3.53%)	2/501 (0.40%)
Sulfamethoxazole–trimethoprim	16/178 (8.99%)	8/110 (7.27%)	8/657 (1.22%)
Amoxicillin-clavulanic	31/131 (23.66%)	9/83 (10.84%)	21/482 (4.36%)

**Table 6:** Annual resistance rates of *Salmonella* spp. isolates to ampicillin and ciprofloxacin, expressed as the number of resistant isolates relative to the total number of tested isolates for each antimicrobial agent.

Antimicrobials	Number of Resistant Isolates/Total Investigated Isolates in the Study Years (%)		
	2023	2024	2025
Ampicillin	39/226 (17.26%)	30/334 (8.98%)	37/356 (10.39%)
Ciprofloxacin	112/219 (51.14%)	178/379 (46.97%)	148/356 (41.57%)

**Table 7:** Romanian studies published between 2016 and 2026 reporting antimicrobial resistance data for bacterial enteropathogens (*Campylobacter* spp., *Salmonella* spp., *Shigella* spp., *Yersinia* spp.) isolated from human cases of acute diarrheal disease. NA = not available.

Enteric Pathogen	Reference(s) (First Author, Year)	Study Period	Number of Human Isolates	Study Design
*Campylobacter* spp.	Baltoiu et al. (2024) [7]	2017–2020	66 *C. jejuni* strains	Molecular surveillance study
	Chiurtu et al. (2025) [31]	Jan 2018–Aug 2024	154 strains	Retrospective study
*Salmonella* spp.	Buzila et al. (2025) [41]	Jan 2024–Aug 2024	109 strains 698 strains 309 strains	Molecular surveillance study
	Sima et al. (2025) [38]	Jan 2018–Dec 2024		Retrospective study
	Gheorghe-Barbu et al. (2025) [46]	2019		Molecular surveillance study
*Shigella* spp.	NA	(-)	(-)	(-)
*Yersinia* spp.	NA	(-)	(-)	(-)

## Data Availability

Data set available on request from the authors.

## Abbreviations

The following abbreviations are used in this manuscript:

AMR	Antimicrobial Resistance
ADD	Acute Diarrheal Disease
FQ	Fluoroquinolones
CIP	Ciprofloxacin
AMP	Ampicillin
SXT	Sulfamethoxazole–trimethoprim
ERY	Erythromycin
TET	Tetracycline
CRO	Ceftriaxone
CTX	Cefotaxime
MIC	Minimum Inhibitory Concentration
